# H172Y mutation perturbs the S1 pocket and nirmatrelvir binding of SARS-CoV-2 main protease through a nonnative hydrogen bond

**DOI:** 10.1101/2022.07.31.502215

**Published:** 2022-08-01

**Authors:** Vinicius Martins de Oliveira, Mohamed Fourad Ibrahim, Xinyuanyuan Sun, Rolf Hilgenfeld, Jana Shen

**Affiliations:** † Department of Pharmaceutical Sciences, University of Maryland School of Pharmacy, Baltimore, Maryland, USA; ‡ Institute of Molecular Medicine, University of Lübeck, Lübeck, Germany; ¶ German Center for Infection Research (DZIF), Hamburg – Lübeck – Borstel – Riems Site, University of Lübeck, Lübeck, Germany

## Abstract

Nirmatrelvir is an orally available inhibitor of SARS-CoV-2 main protease (Mpro) and the main ingredient of PAXLOVID, a drug approved by FDA for high-risk COVID-19 patients. Although the prevalent Mpro mutants in the SARS-CoV-2 Variants of Concern (e.g., Omicron) are still susceptible to nirmatrelvir, a rare natural mutation, H172Y, was found to significantly reduce nirmatrelvir’s inhibitory activity. As the selective pressure of antiviral therapy may favor resistance mutations, there is an urgent need to understand the effect of the H172Y mutation on Mpro’s structure, function, and drug resistance. Here we report the molecular dynamics (MD) simulations as well as the measurements of stability, enzyme kinetics of H172Y Mpro, and IC_50_ value of nirmatrelvir. Simulations showed that mutation disrupts the interactions between the S1 pocket and N terminus of the opposite protomer. Intriguingly, a native hydrogen bond (H-bond) between Phe140 and the N terminus is replaced by a transient H-bond between Phe140 and Tyr172. In the ligand-free simulations, strengthening of this nonnative H-bond is correlated with disruption of the conserved aromatic stacking between Phe140 and His163, leading to a partial collapse of the oxyanion loop. In the nirmatrelvir-bound simulations, the nonnative H-bond is correlated with the loss of an important H-bond between Glu166 and nirmatrelvir’s lactam nitrogen at P1 position. These results are consistent with the newly reported X-ray structures of H172Y Mpro and suggest a mechanism by which the H172Y substitution perturbs the S1 pocket, leading to the decreased structural stability and binding affinity, which in turn explains the drastic reduction in catalytic activity and antiviral susceptibility.

## Introduction

The COVID-19 pandemic is still ongoing and remains a major global health threat. At the end of 2021, the U.S. Food and Drug Administration (FDA) issued an Emergency Use Authorization for Pfizer’s PAXLOVID to treat mild-to-moderate COVID-19 cases.^1,2^ In a recent clinical trial for high-risk non-hospitalized adults with COVID-19,^3^ PAXLOVID reduced the risk of progression to severe disease by 89% as compared to placebo. This antiviral drug is a ritonavir-boosted formulation of nirmatrelvir (PF-07321332), an orally available inhibitor of the SARS-CoV-2 main protease (Mpro). Mpro, which is also known as 3CLpro or Nsp5, is a cysteine protease essential to the viral replication process as it cleaves the majority of the polyproteins pp1a and pp1ab.^4^ Nirmatrelvir is a reversible covalent peptidomimetic inhibitor, which binds to the active site of Mpro and inhibits its proteolytic activity.^5^ Although Mpro is one of the most conserved proteins among coronaviruses,^4^ the rapid and constant evolution of the viral genome raises great concern of potential emergence of antiviral resistance. Several biochemical studies, however, showed that the prevalent Mpro mutants in the Variants of Concern or Variants of Interest declared by the World Health Organization (WHO), such as G15S (Lambda), K90R (Beta), and P132H (Omicron), are still susceptible to nirmatrelvir, with IC50 values and catalytic efficiencies similar to the wild type (WT) Mpro.^6–8^ Nevertheless, biochemical assays of several infrequent natural substitutions, e.g., H164N, H172Y, and Q189K, are associated with reduced activities of nirmatrelvir, among which H172Y caused the largest reduction in the inhibitory activity, with a 233-fold increase in the *K*_*i*_ value of nirmatrelvir according to a disclosure by Pfizer.^1^ Although H172Y is a rare mutation (found in only a few entries of the database GISAID^9^), it may become favored in the future under the selection pressure of nirmatrelvir therapy. Thus, understanding the antiviral resistance mechanism is important and urgently needed.

Motivated by the aforementioned need, we investigated the H172Y mutation effect on the Mpro’s conformation and binding interactions with nirmatrelvir using all-atom molecular dynamics (MD) simulations. As an experimental structure of H172Y Mpro was unavailable at the start of the study (see later discussion), the simulations were initiated from a mutant model. The simulations showed that the H172Y substitution disrupts the interactions between the S1 pocket and N terminus of the opposite protomer. Importantly, the formation of a nonnative hydrogen bond (H-bond) between Tyr172 and the conserved Phe140 is correlated with a partial collapse of the S1 pocket in the free enzyme and the loss of an important H-bond with nirmatrelvir. These results offer a mechanism for the reduced inhibitory activity of nirmatrelvir and also suggest that the H172Y mutation may decrease the catalytic efficiency and dimer stability of the Mpro. Following the simulation study, we have obtained the enzyme kinetic data of H172Y Mpro, which confirmed the reduced catalytic efficiency. The simulation findings regarding the loss of N-finger interactions and formation of a nonnative hydrogen bond corroborate with a recent bioRxiv paper^10^ which reports a free and ligand-bond X-ray structures of H172Y Mpro.

## Results and Discussion

### Simulations of free H172Y Mpro revealed perturbed S1 pocket and N terminus.

To build a structure model of H172Y Mpro for initiating MD simulations, we used the X-ray crystal structure of WT Mpro in complex with nirmatrelvir (PDB id 7vh8, resolution 1.58 Å)^5^ as a template in the homology modelling software Modeller.^11^ Our previous simulation work^12^ showed that His172 is predominantly neutral at physiological pH, and changing to the doubly protonated charged state results in a partial collapse of the S1 pocket. Thus, we rationed that substitution of a neutral histidine with tyrosine would not cause a significant change in the Mpro structure. Indeed, the modeled H172Y Mpro structure is nearly superimposable with the WT, except for a slight displacement of the backbone of Phe140, resulting in a 0.3 Å larger distance between the backbone carbonyl oxygen of Phe140 and the amino nitrogen of Ser1* (asterisk indicates the opposite protomer). Starting from this modeled structure, we carried out MD simulations of the ligand-free as well as the nirmatrelvir-bound H172Y Mpros using the Amber20 program.^13^ Each simulation lasted 2 *μ*s and was repeated once (Table S1). The protonation states were the same as in our previous study of the WT Mpro.^12^ As a control and reference, the ligand-free and nirmatrelvir bound WT Mpros were also simulated using the same settings.

**Figure 1:**
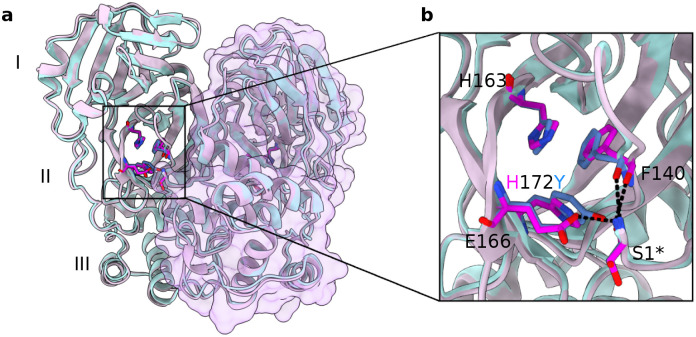
Overlay of the WT and modeled H172Y Mpro structures. **a.** Overlay of the modeled structure of H172Y mutant (blue) on the X-ray structure of WT SARS-CoV-2 Mpro dimer (cyan, PDB id 7vh8).^5^ I, II, and III represent the three domains that compose the Mpro monomer. A surface rendering is shown for protomer B, and inhibitor is hidden. **b.** A zoomed-in view of the S1 pocket and the region of the H172Y mutation. Dashed lines represent conserved hydrogen bonds or electrostatic interactions involving Phe140, Glu166, and Ser1*.

**Figure 2:**
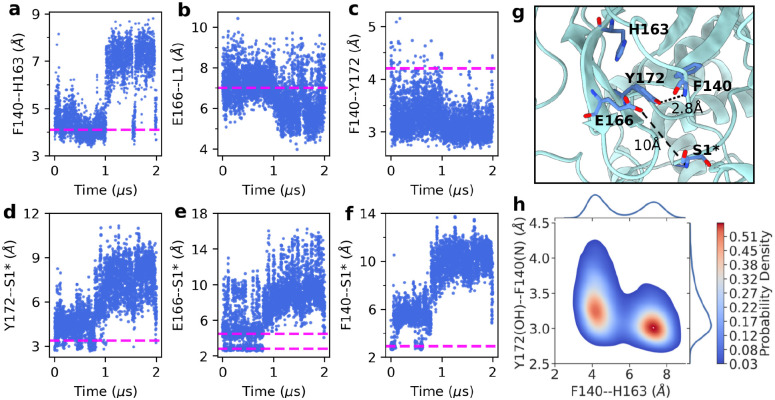
The S1 pocket and N-finger conformation are perturbed in the simulation of the free H172Y SARS-CoV-2 Mpro. **a-f.** Distances as a function of simulation time: **a.** between the center of mass (COM) of the aromatic rings of Phe140 and His163; **b.** between the COM of the carboxylate oxygens of Glu166 and the C*α* atoms of L1; **c.** between the backbone amide nitrogen of Phe140 and the hydroxyl oxygen of Tyr172; **d.** between the hydroxyl oxygen of Tyr172 and the N-terminus nitrogen*; **e.** between the nearest carboxylate oxygen of Glu166 and the N-terminus nitrogen*; **f.** between the backbone carbonyl oxygen of Phe140 and the N-terminus nitrogen*. The magenta dashed lines represent the corresponding average distances sampled by the simulations of the free WT Mpro. In **c.** and **d.**, the magenta lines refer to the distances involving the imidazole nitrogen of H172. **g.** A representative structure of H172Y Mpro taken from the clustering analysis of the last 1-*μ*s trajectory. **h.** Probability density as a function of the F140–H163 and F140–Y172 distances from the last 1-*μ*s trajectory. Analysis here uses the data of protomer A from the simulation run 1. Analysis of protomer B of simulation run 1 and simulation run 2 is given in Fig. S5 and S6, respectively.

The Mpro’s S1 pocket conformation is upheld by several key interactions, one of which is the aromatic stacking between the absolutely conserved Phe140 and His163 (Fig. 1). This interaction is stable in the present WT simulation and the previous^12^ WT simulations started from a different crystal structure (PDB id 6Y2G).^4^ The center-of-mass (COM) distance is about 4 Å between the aromatic rings of Phe140 and His163 (Fig. 2a, magenta line). However, while the stacking interaction was stable in simulation run 2 of the H172Y mutant, it got lost after about 1 *μ*s in simulation run 1, with the F140–H163 stacking distance increased to above 7 Å (Fig. 2a). The breakage of the aromatic stacking is concurrent with a sudden *~*1-Å increase in the heavy-atom root-mean-square deviation (RMSD) of the oxyanion loop (L1, residues 139–145) which harbors Phe140 (Fig. S3), and a sudden *~*2-Å decrease in the COM distance between L1 and Glu166 sidechain. The latter is reminiscent of the L1 collapse observed in the simulations of the H172 protonated WT Mpro^12^ as well as an X-ray structure of SARS-CoV Mpro determined at pH 6 (PDB id 1uj1).^14^

The S1 pocket conformation as well as the stability of the Mpro dimer are also supported by the interactions with the so-called N-finger (residues 1–9) of the opposite protomer.^14^ In particular, Ser1* plays an important role, as its backbone and sidechain maintain H-bond or salt bridge with three absolutely conserved residues in the S1 pocket, His172, Glu166, and Phe140, according to the X-ray structures and the previous^12^ as well as the current WT Mpro simulations. We first consider the H-bond between the hydroxyl group of Ser1* and the imidazole group of His172, which was stable in the WT simulation (Fig. 2d, magenta line). Interestingly, to compensate for the missing imidazole group, the hydroxyl group of Tyr172 initially interacted with the amine nitrogen of Ser1* in both runs of H172Y Mpro (Fig. 2d, Fig. S5 and S6). However, the Tyr172–Ser1* interaction was completely lost after about 0.75 *μ*s in run 1 and 1.8 *μ*s in run 2. The distance between the hydroxyl oxygen and amine nitrogen increased to 5–10 Å in both protomers of run 1 (Fig. 2d) and 14–18 Å in protomer B of run 2 (Fig. S6).

Another prominent N-terminus interaction is with Glu166. Glu166 and the terminal amine form either a H-bond/salt bridge or loose electrostatic interaction in the WT Mpro simulation, with the minimum charge-center distance of 2.5 or 5 Å, respectively (Fig. 2e, magenta lines). However, the Glu166–Ser1* interaction was completely lost after 0.75 *μ*s in run 1 or 1.8 *μ*s in run 2 of the H172Y mutant simulation. The minimum distance between the carboxylate oxygen and amine nitrogen increased to 7–15 Å for both protomers in run 1 (Fig. 2e and Fig. S5) or 17–25 Å for protomer B in run 2 (Fig. S6). Finally, the terminal amine group also maintains a H-bond with the backbone carbonyl group of Phe140 in the WT Mpro simulation (Fig. 2f, magenta line). This interaction was also completely abolished in the simulations of H172Y, whereby the Phe140:O–S1*:N distance increased to over 9 Å for both protomers in run 1 (Fig. 2f and Fig. S5) and over 18 Å in protomer B of run 2 (Fig. S6).

### Formation of a nonnative H-bond disrupts the S1 pocket and nirmatrelvir binding.

A curious question is why the H172Y mutation perturbs the S1 pocket conformation and its interactions with the N-finger. In comparing the H172Y and WT trajectories, we noticed that the hydroxyl group of Tyr172 can occasionally accept a H-bond from the backbone amide nitrogen of Phe140 (Fig. 2c, Fig. 2g, Fig S5, and Fig. S6), whereas the analogous H-bond between the imidazole of His172 and the carbonyl of Phe140 is not possible (Fig. 2c and Fig. 2g). Interestingly, around the same time as the Phe140–His163 stacking got lost in the simulation run 1 of H172Y Mpro, the distance between the hydroxyl oxygen of Phe140 and the amide nitrogen of Phe140 suddenly decreased (Fig. 2c), which resulted in a significant increase of the H-bond occupancy from about 10% to about 50% (Fig. S7). A representative structure (cluster centroid) obtained from clustering analysis of the second half of the trajectory confirms a perturbed S1 pocket, whereby the Phe140–His163 stacking is abolished and Ser1* is moved away from the S1 pocket; however, Tyr172 is in a tight H-bond with the backbone of Phe140. These data suggest that the nonnative H-bond between Tyr172 and Phe140 plays a key role in perturbing the conformational dynamics of the S1 pocket and its interactions with the N-finger. The interaction between Phe140 and Tyr172 appears to compete with the Phe140–Ser1* H-bond and facilitates its breakage.

We further hypothesized that a strong Tyr172–Phe140 H-bond would disrupt the aromatic stacking between Phe140 and His163. To test this hypothesis, we calculated the two-dimensional probability densities for the distances between Phe140 and His163 and between Tyr172 and Phe140. The density map shows a maximum located around the Phe140–His163 and Tyr172–Phe140 distances of 7.5 Å and 3.0 Å, respectively (Fig. 2h), representing a perturbed state in which the Phe140-His163 stacking is disrupted but a stable H-bond between Tyr172–Phe140 is formed. The density map also shows a local density maximum located at the Phe140–His163 and Tyr172–Phe140 distances of 4 Å and 3.1–3.6, respectively (Fig. 2h), representing a state in which the Phe140-His163 stacking interaction is intact and an occasional H-bond is formed between Tyr172 and Phe140. This analysis demonstrates that the formation of the Tyr172–Phe140 H-bond is correlated with the loss of the aromatic stacking between Phe140 and His163, which may be responsible for the partial collapse of the oxyanion loop.

The mutation induced perturbation of the S1 pocket may explain the 233-fold decrease in the nirmatrelvir activity.^2^ To test this hypothesis, we performed two replicas of 2-*μ*s simulation of the WT and H172Y Mpros in complex with nirmatrelvir (Table S1). To facilitate possible conformational changes, the noncovalent binding mode captured by the X-ray structure (PDB id 7vh8)^5^ was used to initiate the simulations. Although the aromatic stacking between Phe140 and His163 was intact in these simulations, the interactions between Ser1* and Glu166 or Phe140 were lost in both simulation runs (Fig. S8 and S9), and the RMSD of the oxyanion loop was unstable (Fig. S3c and S3d). These data are consistent with the simulations of the free H172Y Mpro, and suggest that the S1 pocket is perturbed by the mutation.

In both simulation runs of the inhibitor-bound H172Y Mpro, nirmatrelvir remained bound to H172Y Mpro, although in run 2, the RMSD briefly increased by as much as 1 Å in protomer B (Fig. S10d). The distribution of the RMSD of nirmatrelvir relative to the X-ray structure is broader and the peak is slightly shifted to larger value in the H172Y than the WT simulations, which indicates that nirmatrelvir is more flexible and has a small conformational change when complexed with the mutant Mpro (Fig. S11a). Examination of the protein-ligand interactions showed that the change mainly affects the *γ*-lactam ring in the P1 position, of which the amide nitrogen is in a H-bond with the carboxylate oxygen of Glu166 in the X-ray structure (PDB id 7vh8).^5^ This H-bond was stable in the WT simulation, with an occupancy over 60% (Fig. S11b), but it was significantly weakened in the simulation of the H172Y mutant, with an occupancy about 20% (Fig. S11b). By contrast, the H-bond between the lactam nitrogen and the backbone carboxyl oxygen of Phe140 was stabilized in the mutant simulations, with an occupancy of about 70% as compared to 40% in the WT simulation (Fig. S11b).

**Figure 3:**
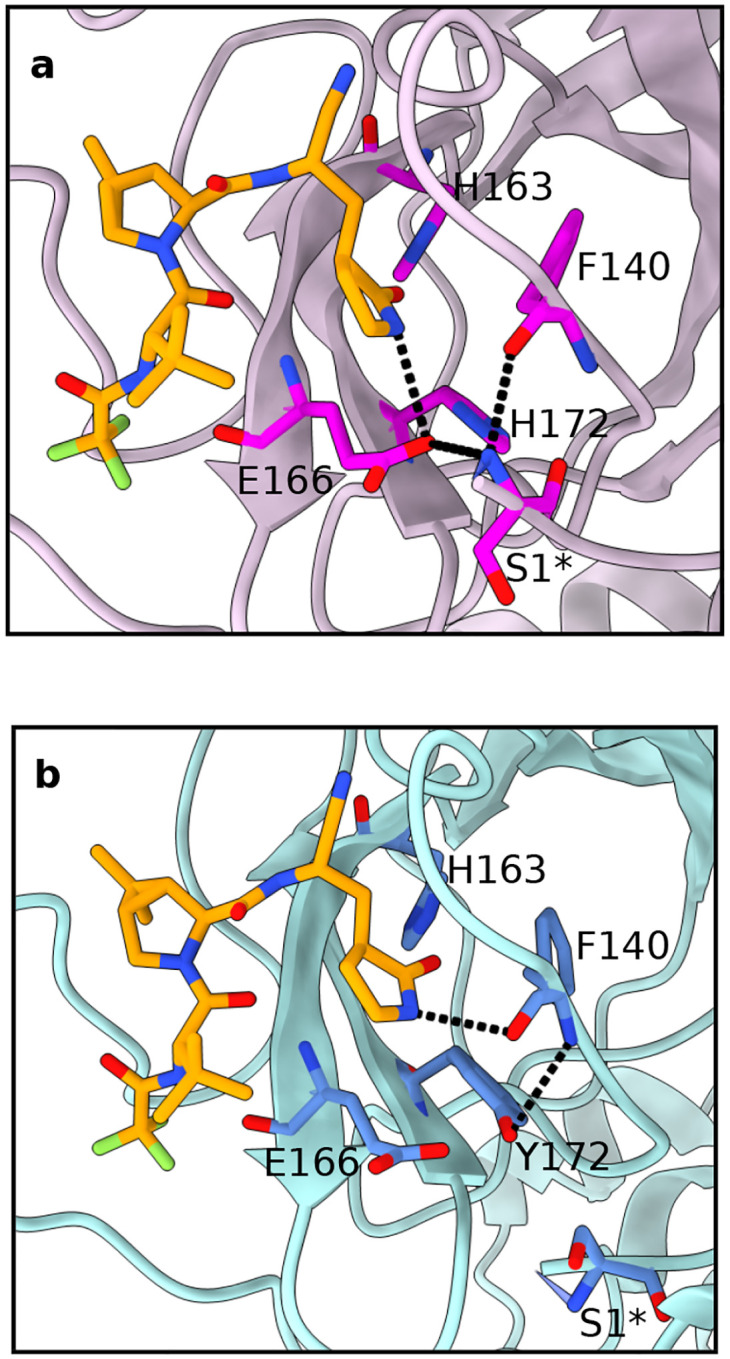
H172Y mutation perturbs the interactions between nirmatrelvir and Mpro. Zoomed-in view of the nirmatrelvir (brown) binding site in the representative structures of WT (**a**) and H172Y (**b**) Mpros from the simulations. The H-bonds that are significantly perturbed by the H172Y mutation are indicated by dashed lines. The relevant S1 pocket residues are shown and labeled. The protomer A’s of the cluster centroids from the clustering analysis of the simulation run 1 of the nirmatrelvir bound WT and H172Y Mpros are used.

The representative structures of the nirmatrelvir bound WT and H172Y Mpros confirmed the mutation-induced change of the P1 site, i.e., the H-bond between the lactam ring and Glu166 is absent (Fig. 3). Importantly, similar to the ligand-free simulations, they also display the nonnative H-bond between Tyr172 and Phe140 as well as the departure of Ser1* from the S1 pocket (Fig. 3). This consistency suggests that the perturbation of the S1 pocket by the H172Y mutation is responsible for the change in the P1 site binding, which we speculate may contribute to the decreased affinity for nirmatrelvir.

### H172Y mutation reduces Mpro’s stability, catalytic activity, and susceptibility to nirmatrelvir.

**Figure 4:**
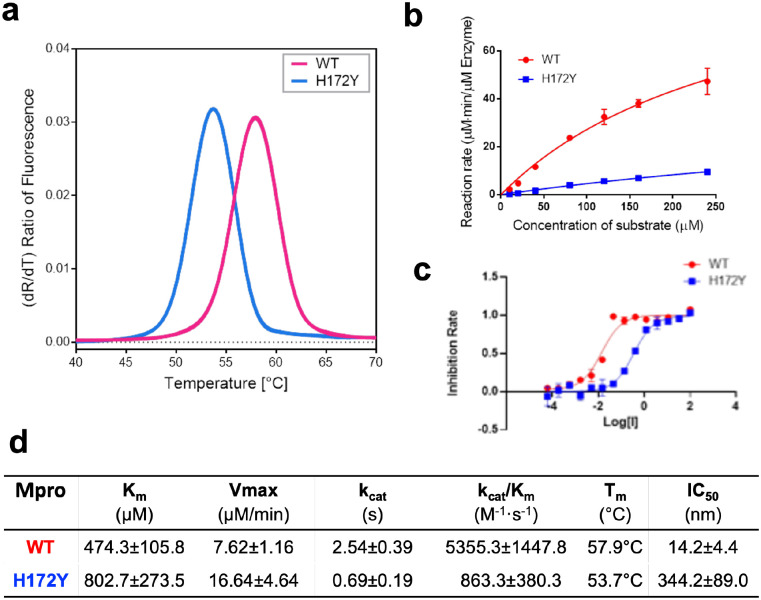
H172Y Mpro has reduced stability, enzyme activity, and susceptibility to nirmatrelvir as compared to the WT. **a.** Melting curves of the WT (red) and H172Y (blue) Mpros based on the temperature profile of the first derivative of the ratio of the autofluorescence at 350 and 330 nm. **b.** Reaction rate vs. substrate concentration for the WT (red) and H172Y (blue) Mpros using the FRET assay. **c.** Inhibition rate of nirmatrelvir vs. its concentration for the WT (red) and H172Y (blue) Mpros. **d.** Summary of the kinetic constants, melting temperatures of the Mpros, and the IC_50_ values of nirmatrelvir.

Following the simulation study, we determined the thermal stability and enzyme kinetics of WT and H172Y Mpros as well as the IC_50_ values of nirmatrelvir (Fig. 4). Thermal-shift assays were used to determine the unfolding temperatures (T_m_) of the WT Mpro and its H172Y mutant (Fig. 4a). The T_m_ for the WT was found to be 57.9 °C, whereas that of the H172Y mutant was lower by 4.2°C (Fig. 4d). This is consistent with the MD results of the MD simulations, which reveal considerable disturbance of the structure.

Kinetic analysis revealed a significant decline in enzymatic activity of H172Y compared to WT Mpro (Fig. 4a). The K_m_ value obtained for the H172Y Mpro was found to be 802.7±273.5 *μ*M, which is 69% larger than the value for the WT Mpro, indicating that the H172Y mutation significantly reduces substrate binding affinity. The k_cat_/K_m_ value obtained for the WT enzyme was 5355.3±1447.8 M^−1^s^−1^, while that for H172Y was only 863.3±380.3 M^−1^s^−1^, i.e. only 16% enzyme activity remains in the FRET assay, compared to the WT. The IC_50_ of nirmatrelvir against the H172Y mutant is 344.2±89.0 nM, around 24 times higher than that for the WT protein. In conclusion, even though the H172Y mutant conveys substantial resistance to nirmatrelvir, its catalytic activity is so disturbed that it is unlikely to become a dominant nirmatrelvir resistance mutation.

As we were preparing the manuscript, a bioRxiv paper^10^ was published that reports the X-ray structures of the free and inhibitor-bound H172Y Mpros. In the ligand-free X-ray structure (PDB id: 8d4j),^10^ the salt bridge between Glu166 and the N-terminus* is lost in one protomer, and the H-bond between Phe140 and the N-terminus* is lost in both protomers (Table S2). These data corroborate the simulation finding of the abolished interactions between Phe140/Glu166 and the N-terminus* (Fig. 2e and f). Note, in the inhibitor bound structure (PDB id: 8d4k),^10^ the position of Ser1 is not resolved. As to the interaction between Phe140 and Tyr172, the X-ray structures (PDB ids: 8d4j and 8d4k)^10^ display the Phe140:O–Tyr172:OH distance of 3.5/3.6 in the free enzyme and 3.3/3.3 Å in the ligand-bound form (Table S2). These distances are similar to the average distances sampled by the simulation trajectories when the S1 pocket is stable (Fig. 2, Fig. S6, S8, and S9). In this work,^10^ the k_cat_/K_m_ value of H172Y Mpro was reported as 13.9-fold lower than the WT (compared to the 6.2-fold decrease determined by us), and the K_i_ value of nirmatrelvir was reported as >113.7 fold higher than the WT.

## Conclusions

MD simulations and biochemical experiments were conducted to characterize the structure, dynamics, function, and antiviral susceptibility of H172Y Mpro. Consistent with the newly reported X-ray structure of the free H172Y Mpro (PDB 8d4j),^10^ simulations demonstrated the loss of interactions between the S1 pocket and the N terminus* as well as the nonnative interaction between the backbone of Phe140 and the sidechain of the mutated residue Tyr172. The latter appears to compete with the native H-bond between the backbone of Phe140 and the N terminus*. Intriguingly, one of the simulation trajectories revealed that strengthening of the nonnative Phe140–Tyr172 H-bond is correlated with the breakage of the conserved aromatic stacking between Phe140 and His163, leading to a partial collapse of the oxyanion loop. This correlation is consistent with the X-ray structures of H172Y Mpro (PDB 8d4j and 8d4k)^10^ and the simulation trajectories, in which the aromatic stacking and oxyanion loop are stable while the Phe140–Tyr172 H-bond is negligible. These data suggest a critical role of the nonnative Phe140–Tyr172 interaction in destabilizing the N-finger and S1 pocket of H172Y Mpro. The latter explains the 4.2°C decrease in T_m_, 69% increase in K_m_, and 94% reduction in the k_cat_/K_m_ value as compared to the WT. The nirmatrelvir-bound simulations further demonstrated that the aforementioned nonnative H-bond is correlated with the loss of an important interaction between Glu166 and nirmatrelvir at the P1 position, which may explain the 24-fold increase in the IC_50_ value of nirmatrelvir.

Taken together, our data offer a mechanism in which the H172Y mutation perturbs the N-finger and S1 pocket, leading to the decreased structural stability and binding affinity of the H172Y Mpro. The latter is responsible for the significant reduction in the catalytic activity and susceptibility to nirmatrelvir. Our finding of the altered P1 site interaction has implication for inhibitor optimization to restore antiviral potency. Although the drastic decline in catalytic activity suggests that H172Y may not become a dominant nirmatrelvir resistance mutation, it is conceivable that double or triple mutations in combination with H172Y may emerge to rescue function while maintaining antiviral resistance (e.g., as demonstrated by a recent work^10^). On that note, our work demonstrates the synergy between predictive MD simulations and biochemical experiments in the active surveillance of continually emerging SARS-CoV-2 mutations.

## Materials and Methods

### Computational methods and protocol

The Modeller software^11^ was used to generate an initial structural model of the H172Y mutant of SARS-CoV-2 Mpro, with the X-ray crystal structure of the wild-type (WT) Mpro in complex with nirmatrelvir (PDB id 7vh8)^5^ as a template. Next, the WT and H172Y Mpro were prepared for MD simulations using the LEAP utility of Amber,^13^ with the termini left free. The protein was represented by the Amber ff14SB force field^15^ and water molecules by the TIP3P model.^16^ The protonation states were determined using the GPU-accelerated GBNeck2-CpHMD titration^17^ with asynchronous pH replica exchange^18^ as in the previous work.^12^ The octahedral water box was used to solvate the protein, with a distance of at least 11 Å between the protein heavy atoms and the edges of the box. Sodium and chloride ions were added to neutralize the system and create an ionic strength of 150 mM. For the nirmatrelvir-bound Mpro simulations, the reversible bound model in the X-ray structure was used. The ligand parameters were generated using the general Amber force field (GAFF2)^19^ and the AM1 BCC method was used to derive the charges^20^ All simulations were carried out using Amber20.^13^ First, energy minimization with a harmonic restraint of 100 kcal/mol/Å^2^ on the protein heavy atoms was performed for 10000 steps using the steepest descent algorithm followed by 10000 steps using the conjugate gradient algorithm. Next, the system was heated from 100 K to 300 K using the same harmonic restraint in the canonical ensemble by 1 ns. Five equilibration stages using harmonic forces of 10, 5, 2, 1, and 0.1 kcal/mol/Å^2^ were then performed for 50 ns in the NPT ensemble. The pressure was maintained at 1 atm using the Berendsen barostat with a relaxation time of 0.1 ps, and the temperature was maintained at 300 K using the Langevin thermostat with a collision frequency of 1.0 ps^−1^.^13^ The particle-mesh Ewald^21^ method was used to treat the long-range electrostatics with a grid spacing of 1 Å. A cutoff of 8 Å was used for van der Waals interactions. SHAKE was used to increase the time step to 2 fs. Finally, the production simulations were performed for 2 *μ*s for both the ligand-free and nirmatrelvir-bound WT and H172Y Mpros. Each simulation was repeated once.

### Protein production and characterization

#### Cloning of SARS-CoV-2 Mpro H172Y.

The H172Y mutation was inserted by overlap extension-PCR reaction. A pair of special primers, H172Y_forward (ACTGGTGTATATGCCGGGACGGACT; the underlined sequence corresponds to the mutated H172Y codon) and H172Y_reverse (AGTCCGTCCCGGCATATACACCAGT) were designed. The first PCR reaction was performed to generate two splice fragments containing a 5′ overhang. The WT Mpro coding gene with BamHI and XhoI sites was amplified from the Mpro construct as described previously,^22^ and was used as template. The second PCR joined these two spliced fragments to generate the PCR product encoding the H172Y mutated Mpro including the cleavage sites of the restriction enzymes for cloning into the vector PGEX-6p-1 (GE Healthcare). The amplified PCR product was digested with BamHI and XhoI and ligated into the vector PGEX-6p-1 digested with the same restriction enzymes. The gene sequence of the Mpro was verified by sequencing (MWG Eurofins).

The sequence-verified SARS-CoV-2 Mpro construct was transformed into E. coli strain BL2 (DE3) (Novagen). Transformed clones were pre-cultured at 37°C in 50 mL 1 × YT medium with ampicillin (100 *μ*g/mL) for 3 h, and the incubated culture was inoculated into 4 L 1 × YT medium supplied with 100 *μ*g/mL ampicillin. 0.5 mM isopropyl-D-thiogalactoside (IPTG) was added for induction of the overexpression of the Mpro gene at 37°C when the OD600 reached 0.8. After 5 h, cells were harvested by centrifugation at 9954 × g, 4°C, for 15 min. The pellets were resuspended in 30 mL buffer A (20 mM Tris, 150 mM NaCl, pH 7.8; pH of all buffers was adjusted at room temperature) and then lysed by sonication on ice. The lysate was clarified by ultracentrifugation at 146,682 × g at 4°C for 1 h. The supernatant was loaded onto a HisTrap FF column (GE Healthcare) equilibrated with buffer A. The HisTrap FF column was washed with 150 mL buffer A to remove unspecifically bound proteins, followed by elution using buffer B (20 mM Tris, 150 mM NaCl, 500 mM imidazole, pH 7.8) with a linear gradient of imidazole ranging from 0 mM to 500 mM, 20 column volumes. The fractions containing target protein were pooled and mixed with PreScission protease at a molar ratio of 5:1 and dialyzed into buffer C (20 mM Tris, 150 mM NaCl, 1 mM DTT, pH 7.8) at 4°C overnight, resulting in the target protein with authentic N- and C-termini. The PreScission-treated Mpro was applied to connected GSTtrap FF (GE Healthcare) and nickel columns to remove the GST-tagged PreScission protease, the His-tag, and protein with uncleaved His-tag. The His-tag-free Mpro in the flow-through was concentrated by using Amicon Ultra 15 centrifugal filters (10 kD, Merck Millipore) at 2773 × g, and 4°C. The protein was loaded onto a HiLoadTM 16/600 SuperdexTM 200pg column (GE Healthcare) equilibrated with buffer A. Fractions eluted from the Superdex200 column containing the target protein with high purity were pooled and subjected to buffer exchange (20 mM Tris, 150 mM NaCl, 1 mM EDTA, 1 mM DTT, pH 7.4).

#### Determination of protein stability of SARS-CoV-2 Mpro WT and H172Y by nano differential scanning fluorimetry (nanoDSF).

Thermal-shift assays of SARS-CoV-2 Mpro and its H172Y mutant were carried out using the nanoDSF method as implemented in the Prometheus NT.48 (NanoTemper Technologies). The nanoDSF method is based on the autofluorescence of tryptophan (and tyrosine) residues to monitor protein unfolding. As the temperature increases, the protein will unfold and the hydrophobic residues of the protein get exposed, the ratio of autofluorescence at wavelengths 350 nm and 330 nm will change. The first derivative of 350/330 nm can be used to determine the melting temperature (Tm). 30 *μ*M of WT or mutant protein were diluted in a final volume of 15 *μ*L reaction buffer containing 20 mM HEPES, 120 mM NaCl, 0.4 mM EDTA, 4 mM DTT, 20% glycerol, pH 7.0. Then the proteins were loaded onto Prometheus NT.48 nanoDSF Grade Standard Capillaries (PR-C002, NanoTemper Technologies), the fluorescence signal was recorded under a temperature gradient ranging from 25 to 90°C (incremental steps of 0.5°C min^−1^). The melting curve was drawn using GraphPad Prism 7.0 software; the values of the first derivative of 350/330 nm were displayed on the Y axis. The melting temperature (Tm) was calculated as the mid-point temperature of the melting curve using the PR.ThermControl software (NanoTemper Technologies).

#### Enzyme Assays.

A fluorescent substrate harboring the cleavage site (indicated by *↓*) of SARS CoV-2 Mpro (Dabcyl-KTSAVLQ*↓*SGFRKM-E(Edans)-NH2; GL Biochem) and buffer composed of 20 mM HEPES, 120 mM NaCl, 0.4 mM EDTA, 4 mM DTT, 20% glycerol, 0.5% DMSO, pH 7.0 was used for the inhibition assay. In the fluorescence resonance energy transfer (FRET)-based cleavage assay, the fluorescence signal of the Edans generated due to the cleavage of the substrate by the Mpro was monitored at an emission wavelength of 460 nm with excitation at 360 nm, using a Flx800 fluorescence spectrophotometer (BioTek). Initially, 10 *μ*L of SARS-CoV-2 Mpro WT at the final concentration of 50 nM, or SARS-CoV-2 Mpro H172Y at 400 nM, was pipetted into a 96-well plate containing pre-pipetted 60 *μ*L of reaction buffer. Subsequently, the reaction was initiated by addition of 30 *μ*L of the substrate dissolved in the reaction buffer to 100 *μ*L final volume, at different final concentrations varied from 10 to 320 *μ*M (10, 20, 40, 80, 120, 160, 240, 320 *μ*M). A calibration curve was generated by measurement of varied concentrations (from 0.04 to 6250 nM) of free Edans, with gain 80 in a final volume of 100 *μ*L reaction buffer. Initial velocities were determined from the linear section of the curve, and the corresponding relative fluorescence units per unit of time (ΔRFU/s) was converted to the amount of the cleaved substrate per unit of time (*μ*M/s) by fitting to the calibration curve of free Edans.

Inner-filter effect corrections were applied for the kinetic measurements according to Liu et al.^22^ The fluorescence of the substrate (in RFU) dissolved in 100 *μ*L final volume of reaction buffer at the corresponding concentrations used for the kinetic assay was measured and defined as f (substrate). Afterwards, 1 *μ*L free Edans was added (final concentration: 1 *μ*M) to each well, and the fluorescence reading was taken as f (substrate + Edans). Simultaneously, a reference value (in RFU) was measured with the same concentration of free Edans in 100 *μ*L of reaction buffer, giving f(reference). The inner-filter correction at each substrate concentration was calculated according to the function: corr% = (f (substrate + Edans) − f (substrate)) / f (reference) × 100%. The corrected initial velocity of the reaction was calculated as V = Vo / (corr%), where Vo represents the initial velocity of each reaction.

As saturation could be achieved, kinetic constants (V_max_ and K_m_) were derived by fitting the corrected initial velocity to the Michaelis-Menten equation, *V* = *V*_max_×[*S*]/(*K*_m_+[*S*]), using GraphPad Prism 7.0 software. k_cat_/K_m_ was calculated according to the equation, *k*_cat_/*K*_m_ = *V*_max_/([*E*] × *K*_m_). Triplicate experiments were performed for each data point, and the value was presented as mean ± standard deviation (SD).

#### Determination of the IC_50_ of nirmatrelvir.

The same substrate was employed as for the determination of the enzyme kinetics. The SPARK Multimode Microplate Reader (TECAN) was used to monitor the signal at same emission wavelength and excitation wavelength. The reaction buffer was 20 mM HEPES, 120 mM NaCl, 0.4 mM EDTA, 4 mM DTT, 20% glycerol, pH 7.0, to achieve a final concentration of 2% DMSO which is same as in the enzyme kinetics measurement. Stock solutions of the compounds were prepared with 100% DMSO. For the determination of the IC_50_, 50 nM of SARS-CoV-2 Mpro or 400 nM of SARS-CoV-2 Mpro H172Y was incubated with nirmatrelvir at various concentrations from 0 to 100 *μ*M in reaction buffer at 37°C for 10 min. Afterwards, the FRET substrate at a final concentration of 10 *μ*M was added to each well, at a final total volume of 100 *μ*L, to initiate the reaction. The GraphPad Prism 7.0 software (GraphPad) was used for the calculation of the IC_50_ values. Measurements of inhibitory activity of nirmatrelvir were performed in triplicate and are presented as the mean±SD.

## Supplementary Material

Supplement 1
