# Supplementary material for: H172Y mutation perturbs the S1 pocket and nirmatrelvir binding of SARS-CoV-2 main protease through a nonnative hydrogen bond
Source: bioRxiv. 2022 Aug 1:2022.07.31.502215. Preprint. [Version 1] doi: 10.1101/2022.07.31.502215 (PMC9387124; doi:10.1101/2022.07.31.502215)
Supplement: Supplementary file 1 [file media-1.pdf]

Supporting Information: H172Y mutation perturbs the S1 pocket and nirmatrelvir binding of SARS-CoV-2 main protease through a nonnative hydrogen bond

Vinicius Martins de Oliveira,[†] Mohamed Fourad Ibrahim,[‡] Xinyuanyuan Sun,[¶] Rolf Hilgenfeld,[‡,§] and Jana Shen[*,†]

† *Department of Pharmaceutical Sciences, University of Maryland School of Pharmacy, Baltimore, Maryland, USA*

‡ *Institute for Molecular Medicine, University of Lübeck, Lübeck, Germany*

¶ *Institute For Molecular Medicine, University of Lübeck, Lübeck, Germany*

§ *German Center for Infection Research (DZIF), Hamburg – Lübeck – Borstel – Riems Site, University of Lübeck, Lübeck, Germany*

E-mail: jana.shen@rx.umaryland.edu

List of Tables

S1 Summary of the simulations performed in this work S-4

S2 Summary of relevant distances in the X-ray structure of H172Y Mpro S-4

List of Figures

S1 Probability distributions of the relevant distances involving the S1 pocket in ligand-free WT Mpro S-5

S2 The overall structure of H172Y Mpro was stable in all simulation runs of ligand-free and nirmatrelvir bound H172Y Mpro S-6

S3 Structural stability of the oxyanion loop in the ligand-free and nirmatrelvir bound simulations of H172Y Mpro S-7

S4 Structural stability of the initial part of N-finger in the ligand-free and nirmatrelvir bound simulations of H172Y Mpro S-8

S5 Run 1 of the ligand-free H172Y Mpro: both Ser1* interactions and S1 pocket of protomer B were unstable S-9

S6 Run 2 of the ligand-free H172Y Mpro: S1 pocket was stable but the Ser1* interactions were unstable S-10

S7 Occupancy of the hydrogen bond formed between Phe140 and Tyr172 in run 1 of the ligand-free H172Y Mpro S-11

S8 The Ser1* interactions were disrupted in the simulation run 1 of the nirmatrelvir bound H172Y Mpro S-12

S9 Run 2 of the nirmatrelvir bound H172Y Mpro: the Ser1–S1 pocket interactions were unstable S-13

S10 Nirmatrelvir was stably bound in the WT and H172Y Mpros during the simulations. S-14

S11 Binding interactions between nirmatrelvir and Mpro are affected by the H172Y mutation S-15

Table S1: Summary of the simulations performed in this work

Mpro	PDB ID	Set up	Simulation time
WT	7vh8	ligand-free	2 μs x 2
WT	7vh8	nirmatrelvir bound[a]	2 μs x 2
H172Y	modeled	ligand-free	2 μs x 2
H172Y	modeled	nirmatrelvir bound[a]	2 μs x 2

[a]Simulations started from the X-ray structure (PDB ID: 7vh8),[S1] in which nirmatrelvir was noncovalently bound with the SARS-CoV-2 Mpro. [b]The X-ray structure of H172Y Mpro in complex with the reversible covalent inhibitor 13b-K (Ibraham and Hilgenfeld, in preparation for submission)

Table S2: Summary of relevant distances in the S1 pocket from the X-ray structure of H172Y Mpro[a]

	8d4j	8d4k
F140–H163	3.8/3.8	3.7/3.7
E166–L1	7.6/7.1	7.6/7.6
F140(N)–Y172(OH)	3.5/3.6	3.3/3.3
Y172(OH)–S1*(N)	4.1/4.4	n/d
E166(OE1/2)–S1*(N)	4.3/3.3	n/d
F140(O)–S1*(N)	6.5/6.5	n/d

[a]All distances are in unit Å. F140–H163 refers to the distance between the center-of-mass (COM) of the aromatic rings of Phe140 and His163. E166–L1 refers to the distance between the COM of the carboxylate oxygens of Glu166 and Cα atoms of oxyanion loop. F140–Y172 refers to the distance between the amide nitrogen of Phe140 and the hydroxyl oxygen of Tyr172. Y172–S1* refers to Distance between the hydroxyl group of Tyr172 and the amino nitrogen of Ser1* of the opposite protomer. E166–S1* refers to the distance between the nearest carboxylate oxygen of Glu166 and the amino nitrogen of Ser1* of the opposite protomer. F140–S1* refers to the distance between the backbone carbonyl oxygen of Phe140 and the amino nitrogen of Ser1* of the opposite protomer. In the PDB entry 8d4k, the coordinates of Ser1 are not resolved; thus, distances involving S1 are listed as n/d. The X-ray crystal structures determined by Hu, Wang et al.,[S2] 8d4j: ligand free H172Y Mpro. 8d4k: H172Y Mpro in complex with a covalent inhibitor GC-376.

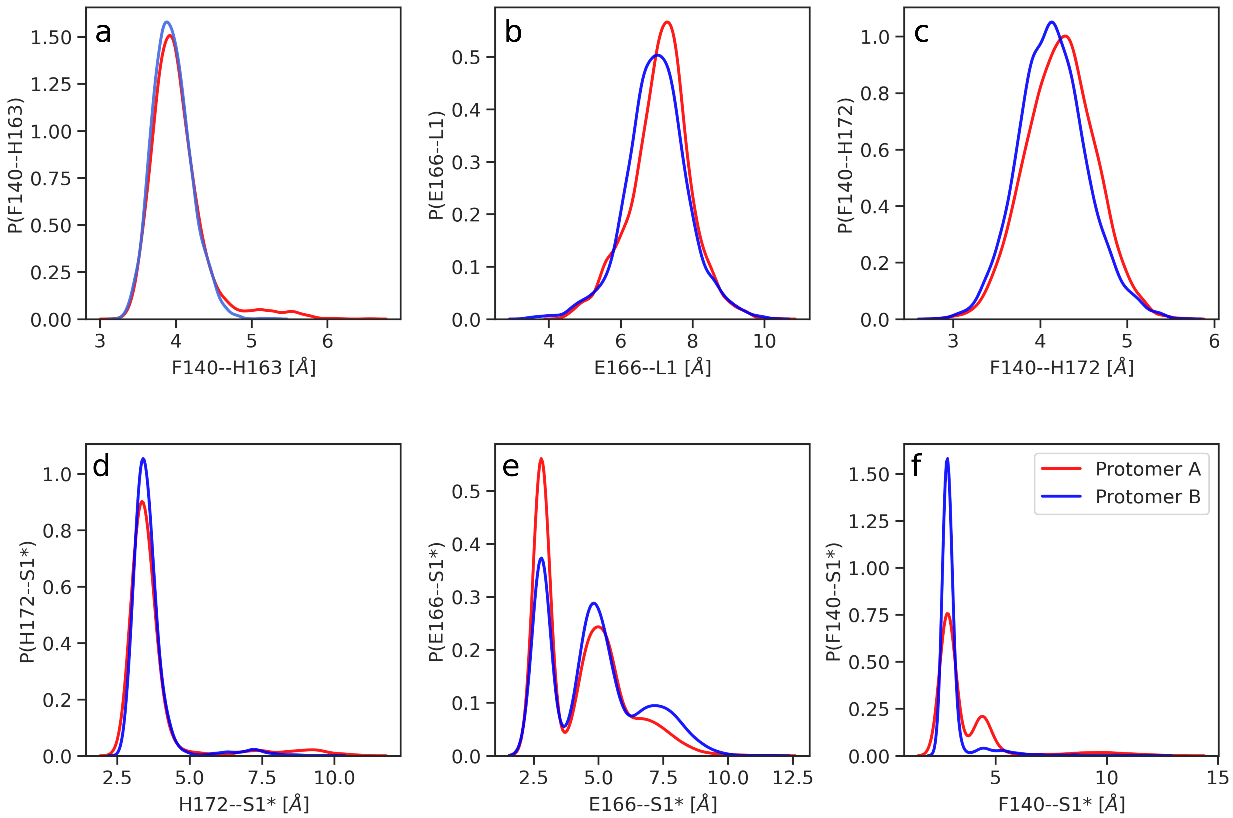

Figure S1: **Probability distributions of distances involving the S1 pocket residues in the ligand-free WT Mpro. The most probable values were used as references in the analysis of the H172Y Mpro simulations.** (a) Probability distribution of the distance between the center-of-mass (COM) of the aromatic rings of Phe140 and His163. (b) Probability distribution of the distance between the COM of the carboxylate oxygens of Glu166 and Cα atoms of residues L1. (c) Probability distribution of the distance between the amide nitrogen of Phe140 and the hydroxyl oxygen of His172. (d) Probability distribution of the distance between the hydroxyl group of His172 and the amino nitrogen of S1*. (e) Probability distribution of the distance between the nearest carboxylate oxygen of Glu166 and the amino nitrogen of Ser1*. (f) Probability distribution of the distance between the backbone carbonyl oxygen of Phe140 and the amino nitrogen of Ser1*. The red and blue curves represent protomer A and B, respectively. The calculations used the data from both simulation runs of ligand-free WT Mpro.

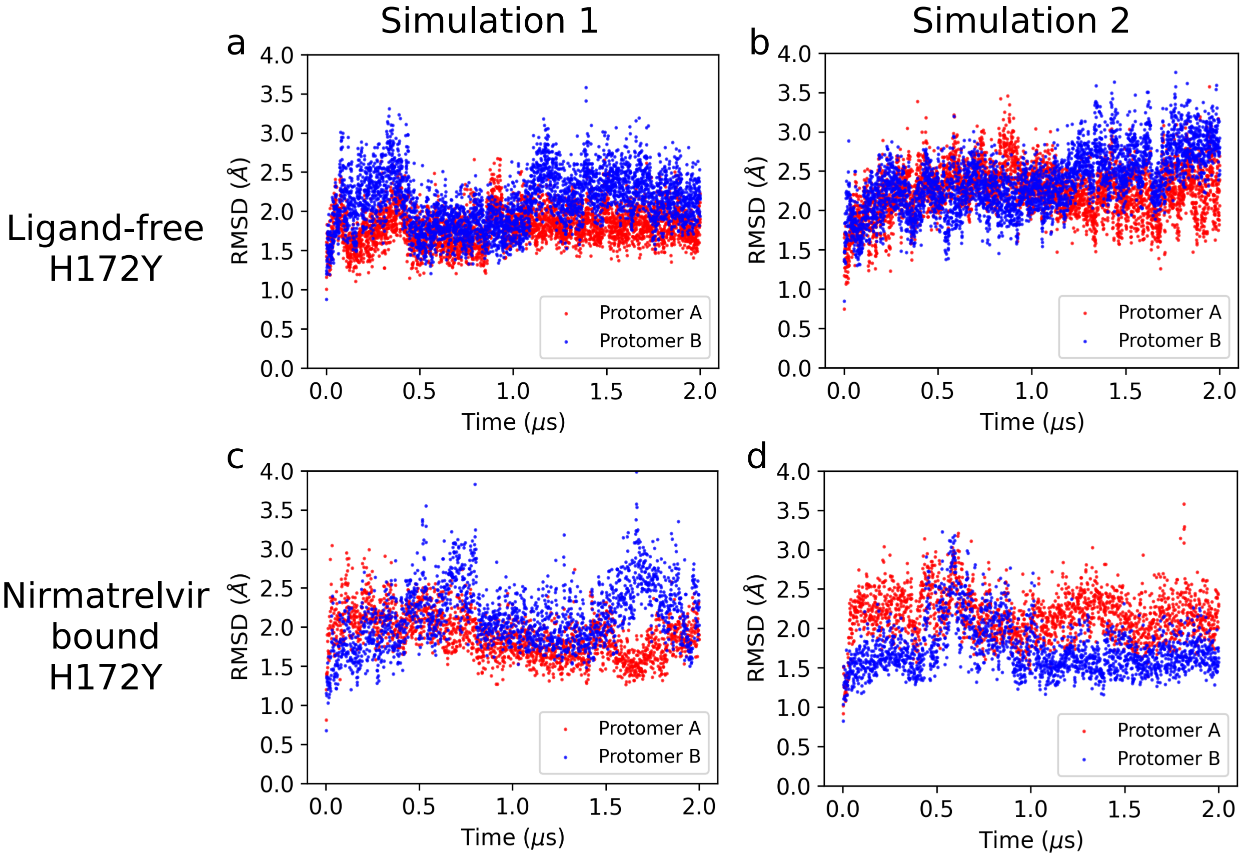

Figure S2: **The overall structure of the H172Y Mpro was stable in all simulation runs of ligand-free and nirmatrelvir bound H172Y Mpro.** Heavy-atom root-mean-square deviation (RMSD) of the ligand-free (a, b) and nirmatrelvir bound (c, d) H172Y Mpros with respect to the mutant model as a function of simulation time. Simulation runs 1 and 2 are shown on the left and right panels, respectively.

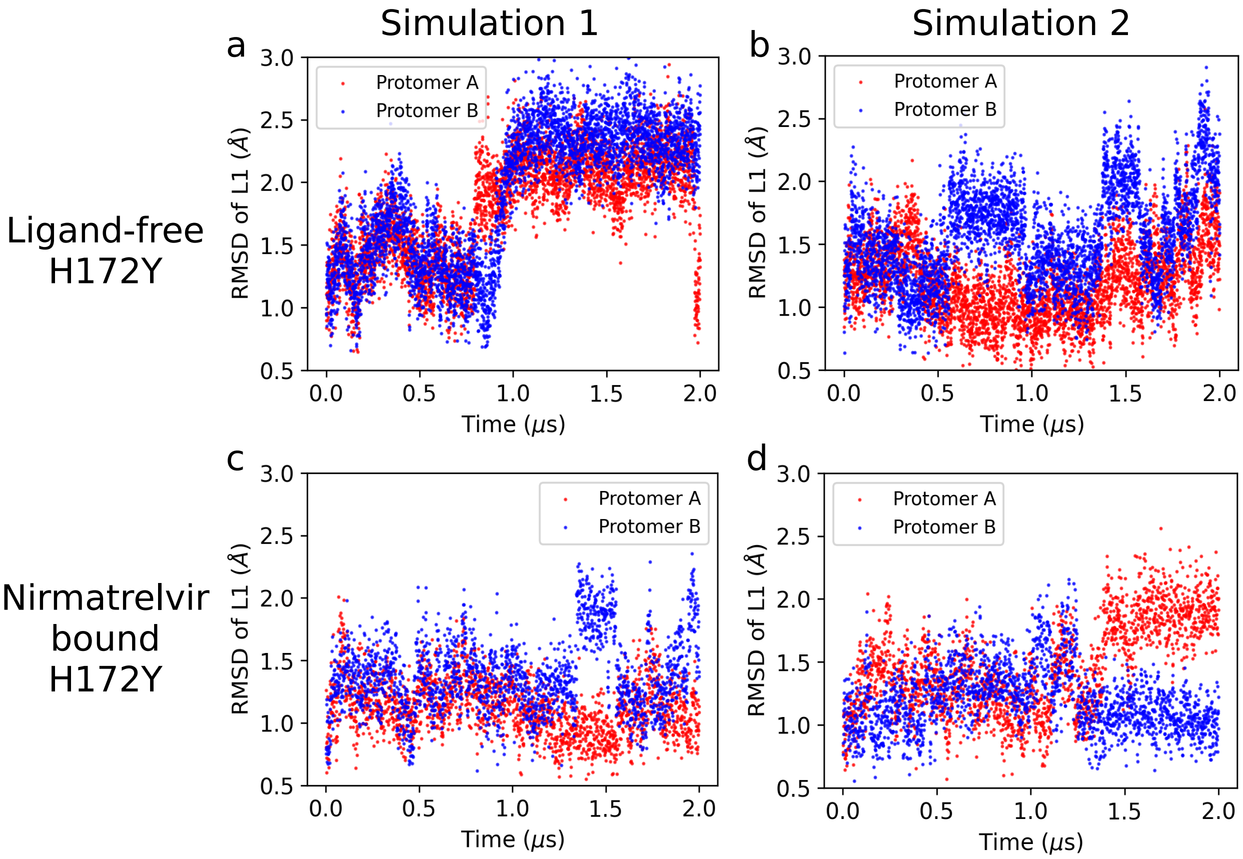

Figure S3: **Structural stability of the oxyanion loop in the ligand-free and nirmatrelvir bound simulations of H172Y Mpro.** Heavy-atom RMSD of the Mpro oxyanion loop (residues 138-145, L1) with respect to the to the mutant model as a function of simulation time in the ligand-free (a, b) and nirmatrelvir bound H172Y (c, d) Mpros. Simulation runs 1 and 2 are shown on the left and right panels, respectively.

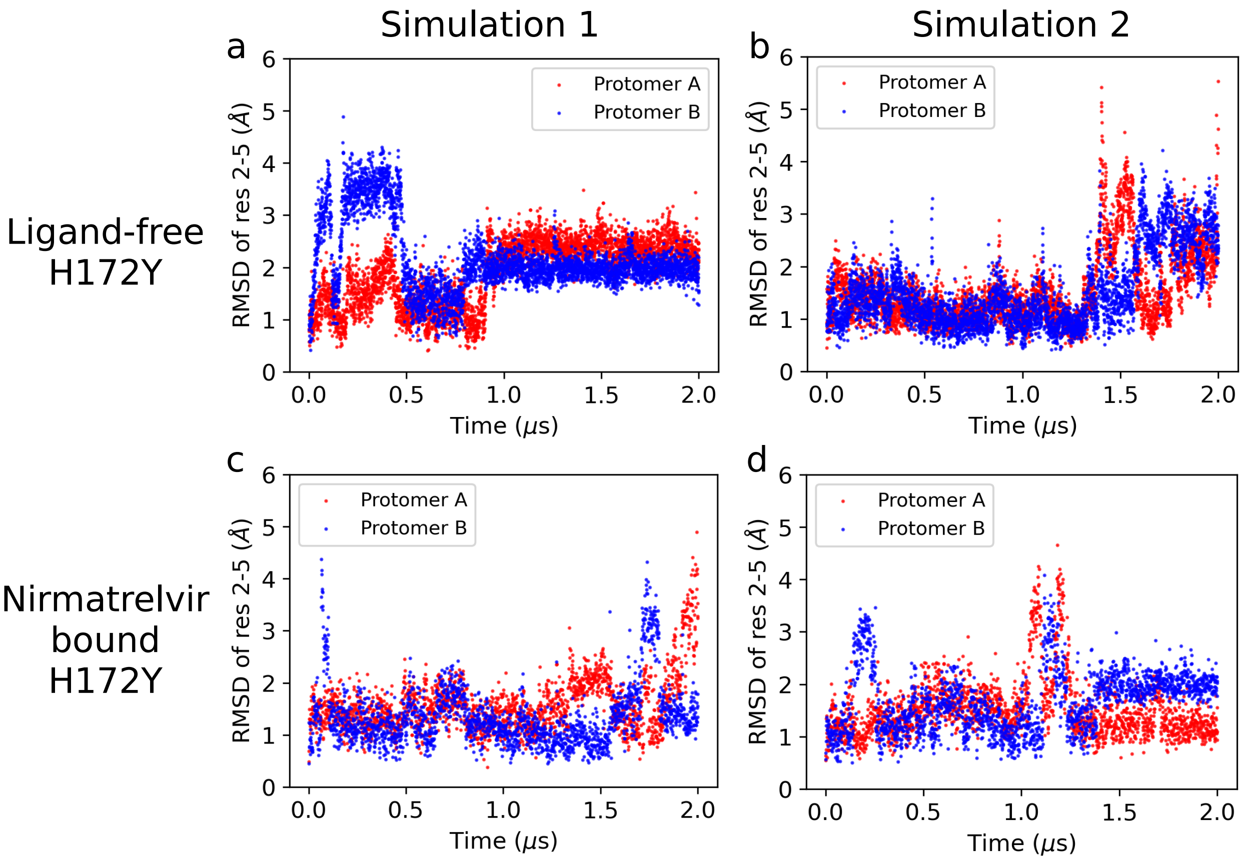

Figure S4: **Structural stability of residues 2-5 in the ligand-free and nirmatrelvir bound simulations of H172Y.** Heavy-atom root-mean-square deviation (RMSD) of the ligand-free (a, b) and nirmatrelvir bound (c, d) H172Y Mpros with respect to the mutant model as a function of simulation time. Simulation runs 1 and 2 are shown on the left and right panels, respectively.

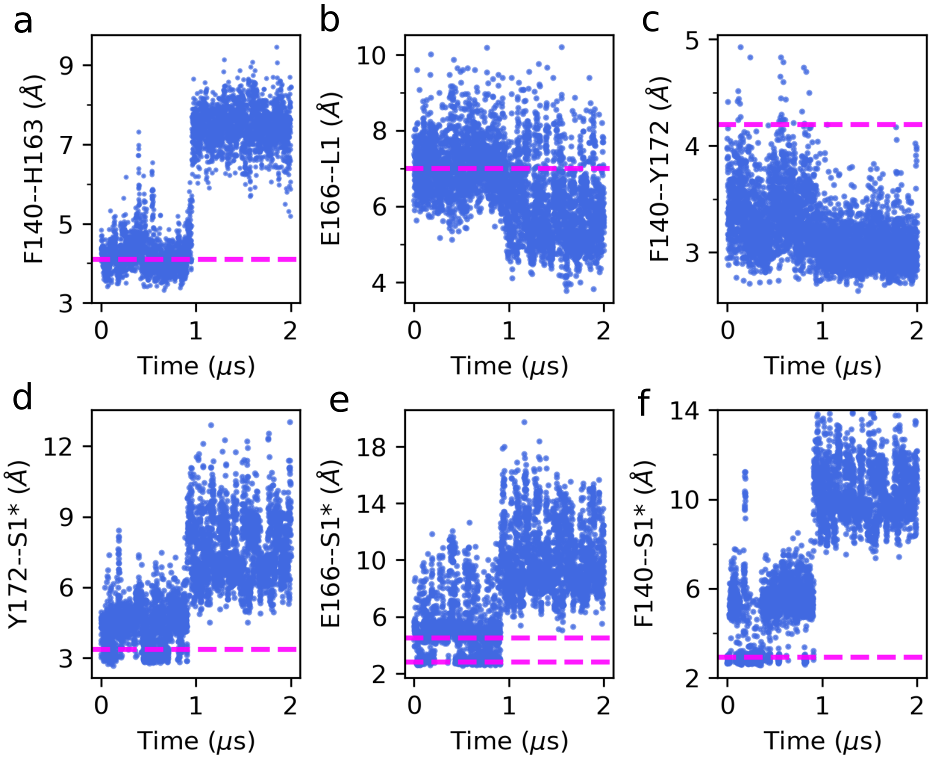

Figure S5: **Run 1 of the ligand-free H172Y Mpro: both Ser1* interactions and S1 pocket of protomer B were unstable.** (a) Distance between the center-of-mass (COM) of the aromatic rings of Phe140B and His163B. (b) Distance between the COM of the carboxylate oxygens of Glu166B and Cα atoms of residues L1. (c) Distance between the amide nitrogen of Phe140B and the hydroxyl oxygen of Tyr172B. (d) Distance between the hydroxyl group of Tyr172B and the amino nitrogen of Ser1A. (e) Distance between the nearest carboxylate oxygen of Glu166B and the amino nitrogen of Ser1A. (f) Distance between the backbone carbonyl oxygen of Phe140B and the amino nitrogen of Ser1A. The magenta dashed lines in the plots represent the average distances sampled in the simulations of the WT Mpro.

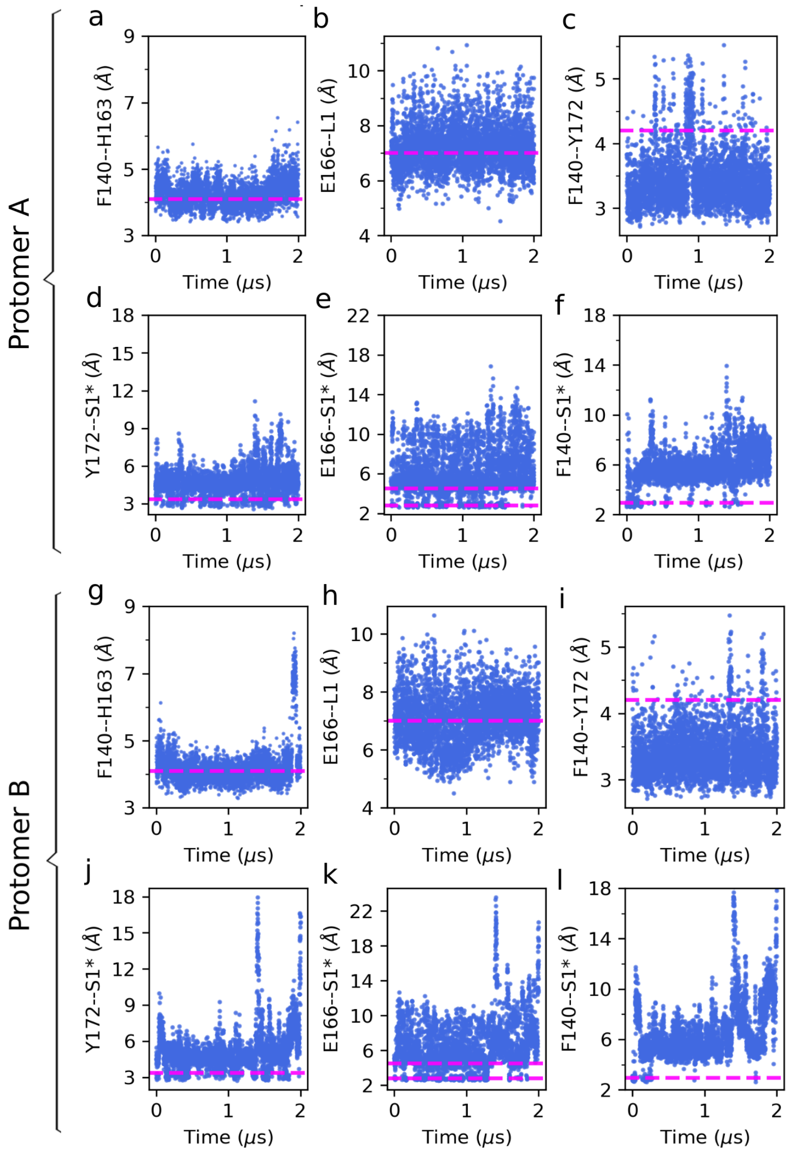

Figure S6: **Run 2 of the ligand-free H172Y Mpro: S1 pocket was stable but the Ser1* interactions were unstable.** (a, g) Distance between the COM of the aromatic rings of Phe140 and His163 in protomer A (a) and B (g). (b, h) Distance between the center of mass (COM) of the carboxylate oxygens of Glu166 and that of the oxyanion loop (Cα atoms of residues 138-145) in protomer A (b) and B (h). (c, i) Distance between the amide nitrogen of Phe140 and the hydroxyl oxygen of Tyr172 in protomer A (c) and B (i). (d, j) Distance between the hydroxyl group of Tyr172 and the N-terminal amino nitrogen of Ser1*. (e, k) Distance between the nearest carboxylate oxygen of Glu166B and the amino nitrogen of Ser1*. (f, l) Distance between the backbone carbonyl oxygen of Phe140 and the N-terminus amino nitrogen of Ser1*. The magenta dashed lines in the plots represent the average distances sampled by WT simulations.

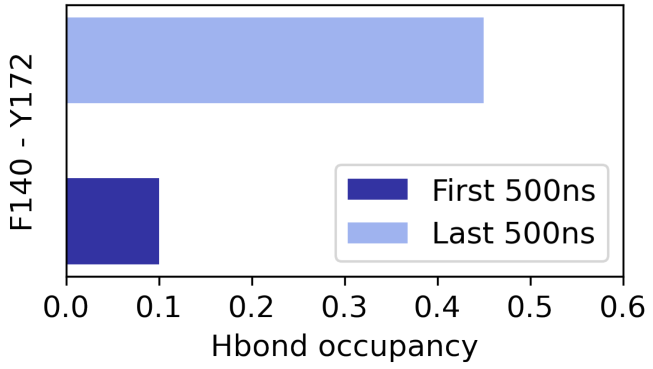

Figure S7: **Run 1 of the ligand-free H172Y Mpro: Occupancy of the hydrogen bond formed between amide nitrogen of Phe140 and the hydroxyl oxygen of Tyr172.** The occupancy was calculated for the first 0.5 μs (dark blue) and last 0.5 μs of the 2-μs simulation.

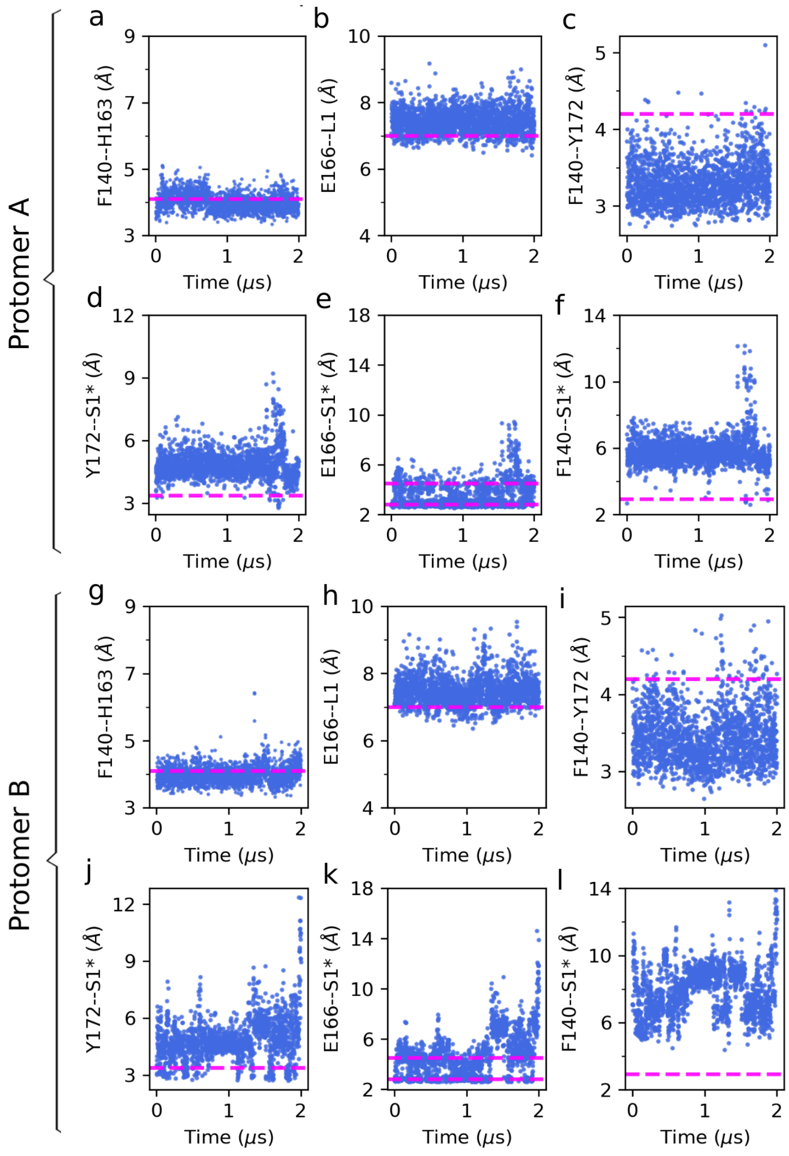

Figure S8: **Run 1 of the nirmatrelvir bound H172Y Mpro: The Ser1* interactions were disrupted.** (a, g) Distance between the COM of the aromatic rings of Phe140 and His163 in protomer A (a) and B (g). (b, h) Distance between the center of mass (COM) of the carboxylate oxygens of Glu166 and that of the oxyanion loop (Cα atoms of residues 138-145) in protomer A (b) and B (h). (c, i) Distance between the amide nitrogen of Phe140 and the hydroxyl oxygen of Tyr172 in protomer A (c) and B (i). (d, j) Distance between the hydroxyl group of Tyr172 and the N-terminus amino nitrogen in the opposite protomer (Ser1*). (e, k) Distance between the nearest carboxylate oxygen of Glu166B and the amino nitrogen of Ser1*. (f, l) Distance between the backbone carbonyl oxygen of Phe140 and the amino nitrogen of Ser1*. The magenta dashed lines in the plots represent the average distances sampled by WT simulations.

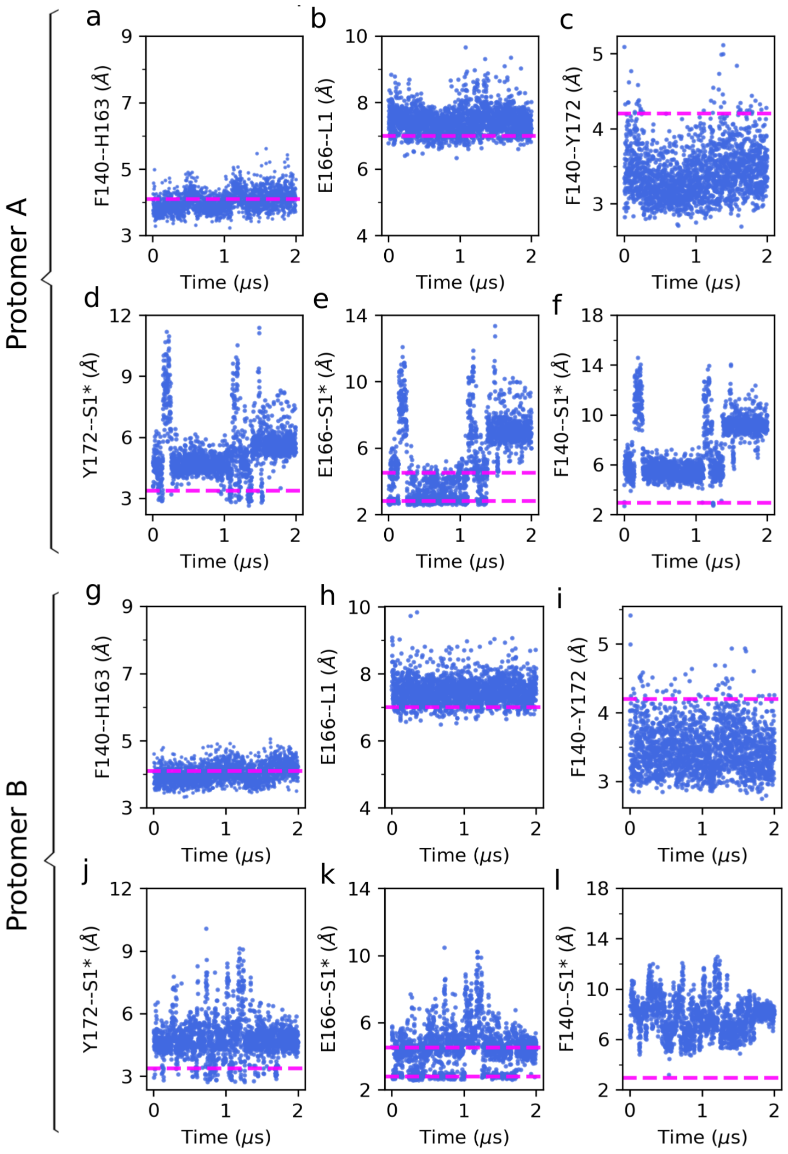

Figure S9: **Run 2 of the nirmatrelvir bound H172Y Mpro: the Ser1–S1 pocket interactions were disrupted.** (a, g) Distance between the COM of the aromatic rings of Phe140 and His163 in protomer A (a) and B (g). (b, h) Distance between the center of mass (COM) of the carboxylate oxygens of Glu166 and that of the oxyanion loop (Cα atoms of residues 138–145) in protomer A (b) and B (h). (c, i) Distance between the amide nitrogen of Phe140 and the hydroxyl oxygen of Tyr172 in protomer A (c) and B (i). (d, j) Distance between the hydroxyl group of Tyr172 and the amino nitrogen of Ser1*. (e, k) Distance between the nearest carboxylate oxygen of Glu166B and the amino nitrogen of Ser1*. (f, l) Distance between the backbone carbonyl oxygen of Phe140 and the amino nitrogen of Ser1*. The magenta dashed lines in the plots represent the average distances sampled by WT simulations.

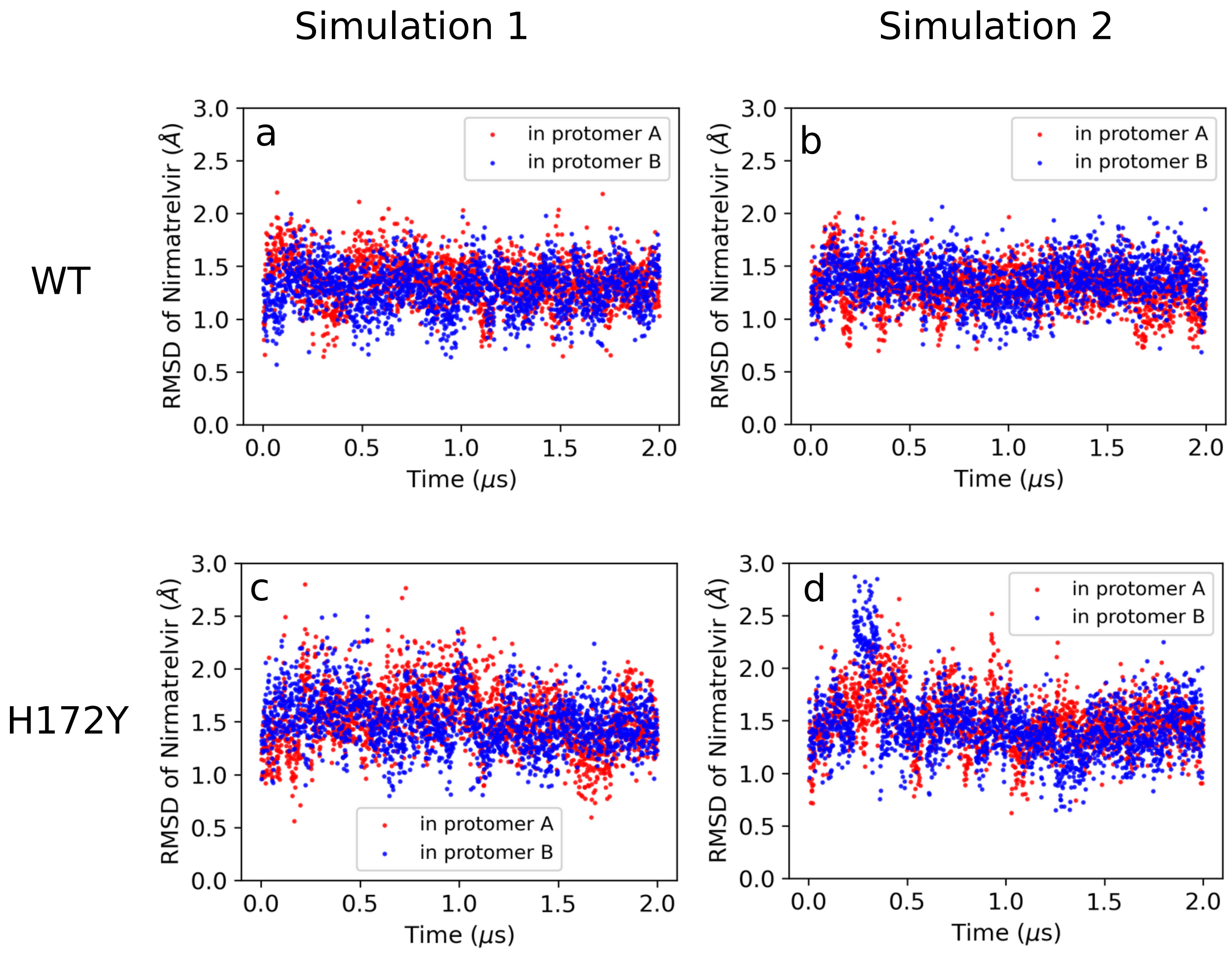

Figure S10: **Nirmatrelvir was stably bound in the WT and H172Y Mpros during the simulations.** (a, b) Time series of the RMSD of nirmatrelvir in the WT Mpro with respect to the crystal structure (PDB id 7vh8) in the simulation run 1 (a) and 2 (b). (c, d) Time series of the RMSD of nirmatrelvir in the H172Y Mpro with respect to the mutant model in the simulation run 1 (c) and 2 (d).

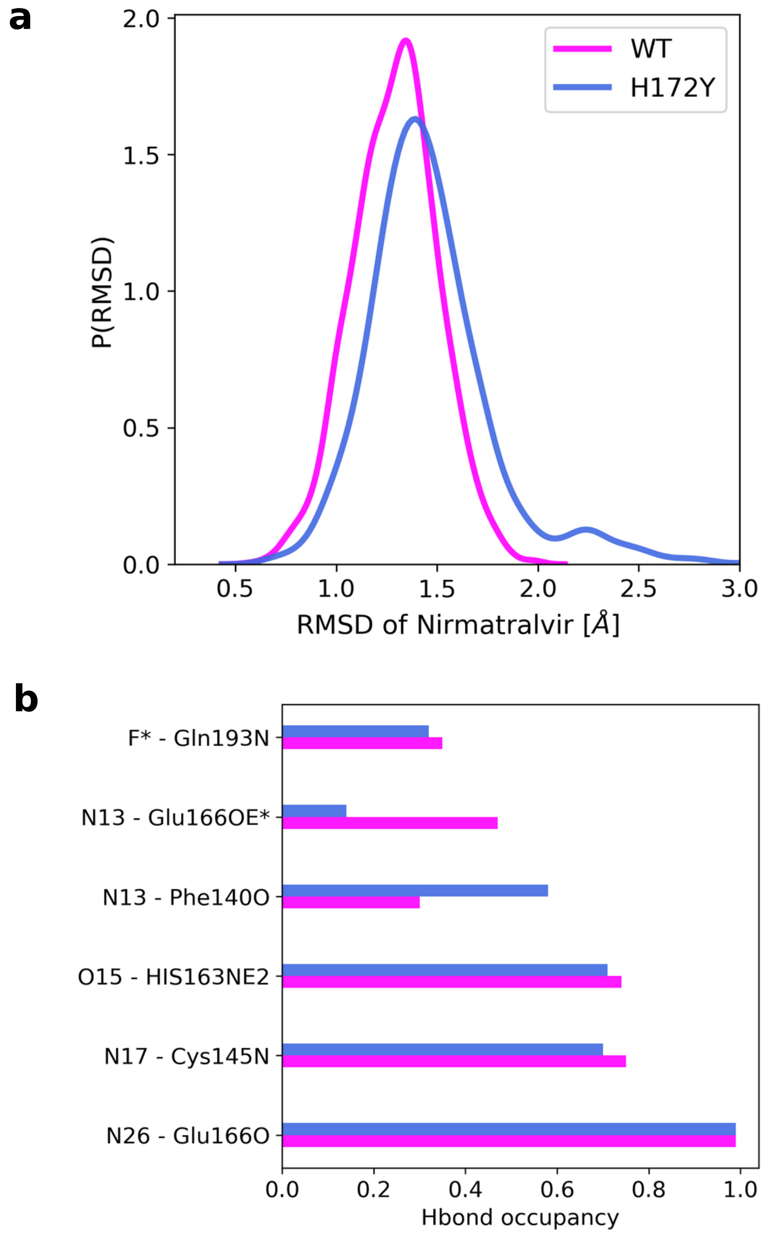

Figure S11: **Binding interactions between nirmatrelvir and Mpro are affected by the H172Y mutation.** (a) Probability distributions of the heavy atom RMSD of nirmatrelvir in the WT (magenta) and H172Y (blue) Mpros with respect to the X-ray structure (PDB id 7vh8). Data from both simulation runs were used. (b) Occupancies of the hydrogen bonds between nirmatrelvir atoms and residues in the WT (magenta) and H172Y (blue) Mpros. The data from the last 1 μs of both simulation runs were used.

References

(S1) Zhao, Y.; Fang, C.; Zhang, Q.; Zhang, R.; Zhao, X.; Duan, Y.; Wang, H.; Zhu, Y.; Feng, L.; Zhao, J.; Shao, M.; Yang, X.; Zhang, L.; Peng, C.; Yang, K.; Ma, D.; Rao, Z.; Yang, H. Crystal Structure of SARS-CoV-2 Main Protease in Complex with Protease Inhibitor PF-07321332. *Protein Cell* **2021**,

(S2) Hu, Y.; Lewandowski, E. M.; Tan, H.; Morgan, R. T.; Zhang, X.; Jacobs, L. M.; Butler, S. G.; Mongora, M. V.; Choy, J. S.; Chen, Y., et al. Naturally occurring mutations of SARS-CoV-2 main protease confer drug resistance to nirmatrelvir. *bioRxiv* **2022**,
